# Comparative Study on Various Authentication Protocols in Wireless Sensor Networks

**DOI:** 10.1155/2016/6854303

**Published:** 2016-01-13

**Authors:** S. Raja Rajeswari, V. Seenivasagam

**Affiliations:** ^1^Department of Computer Science and Engineering, Regional Centre of Anna University, Tirunelveli, Tamil Nadu 627 007, India; ^2^Department of Computer Science and Engineering, National Engineering College, Kovilpatti, Tamil Nadu 628 503, India

## Abstract

Wireless sensor networks (WSNs) consist of lightweight devices with low cost, low power, and short-ranged wireless communication. The sensors can communicate with each other to form a network. In WSNs, broadcast transmission is widely used along with the maximum usage of wireless networks and their applications. Hence, it has become crucial to authenticate broadcast messages. Key management is also an active research topic in WSNs. Several key management schemes have been introduced, and their benefits are not recognized in a specific WSN application. Security services are vital for ensuring the integrity, authenticity, and confidentiality of the critical information. Therefore, the authentication mechanisms are required to support these security services and to be resilient to distinct attacks. Various authentication protocols such as key management protocols, lightweight authentication protocols, and broadcast authentication protocols are compared and analyzed for all secure transmission applications. The major goal of this survey is to compare and find out the appropriate protocol for further research. Moreover, the comparisons between various authentication techniques are also illustrated.

## 1. Introduction 

Wireless sensor networks (WSNs) are rapidly growing in popularity due to the low cost solutions for a variety of challenges in the real-world. WSN has no infrastructure support, is quickly deployed in a region with several low-cost sensor nodes, is employed for monitoring the environment, and is rigid to maintain its security. It comprises huge number of resource sensor nodes, which are spatially dispersed in the hostile environment. The task of the sensor nodes is to sense the physical phenomena from their immediate neighbors and process and transfer the sensed data to the base stations. Multihop communication is preferred in WSN as the number of nodes is very large, and sensor nodes have constraints with respect to power, computation, communication, and storage.

Security in WSN becomes crucial since the nodes after the deployment cannot be manually maintained and observed. This situation becomes a major issue in WSN due to its network of communication. The authentication is provided to the data that can be sent or accessed by any node in the network. Also, it is critical to prevent and gain the information from the unauthorized users. As new threats and attack models are proposed, several kinds of authentication mechanisms have been introduced in WSN security. Authentication mechanism can be differentiated based on the following criteria:

- authenticating unicast, multicast, or broadcast messages,
- symmetric (shared key) or asymmetric (public key) cryptographic method,
- static, mobile, or both aspects of WSN.

Various researches have focused on point-to-point authentication mechanisms, which authenticate unicast messages [1–3] in WSN. In spite of being secure, unicast methods cannot be applied straightly to either multicast or broadcast messages. Broadcast messages are straightly obtained from the reliable sources and cannot be changed during transmission. The basic components of a broadcast authentication process are

- checking the source identity from which the message originates,
- confirming the message integrity for ensuring the message originality.

Additionally, it offers precaution against (a) forgery, (b) replay attacks, and (c) impersonation, which are main features of the authentication mechanisms. There are two authentication mechanisms based on the cryptographic methods as discussed above. It can either be a symmetric method or an asymmetric method. The former methods use shared key cryptography, where both the sender and the receiver employ similar key in the process of authentication and verification. The latter case uses public key cryptography, where the sender signs a message with the private key and the receivers authenticate it by the respective public key.

In this survey, various existing authentication protocols in wireless sensor networks are discussed. A list of major issues and open research challenges are compared and analyzed. Moreover, an exhaustive survey on the available protocols for authentication in the wireless sensor networks and their applications is provided. The survey also contains the major aspects of examining the protocols on the basis of quality measurement as needed for authentication mechanisms. The comparison tables are provided for decision-making on the most appropriate protocols. It fulfils the requirements of the particular application scenario. This paper reviews several authentication protocols in WSN and its major contributions are listed as follows:

- comparison of various authentication protocols,
- information about several existing authentication protocols,
- analyses of various schemes with different parameters in the existing methodologies.

The rest of this paper is organized as follows.  Section 2 reviews the issues of security in the wireless sensor networks.  Section 3 briefly summarizes the definition, procedures, and issues of authentication in the wireless sensor networks.  Section 4 discusses the various authentication protocols in the wireless sensor networks.  Section 5 presents a discussion on various protocols. And finally, the paper concludes with Section 6.

## 2. Security Issues in Wireless Sensor Networks

### 2.1. Threats/Attacks on Sensor Node Routing

Several WSN routing protocols are simple and are vulnerable to attacks from those works on routing in ad hoc networks. Most threats against WSNs fall into one of the following groups:

- spoofed, altered, or replayed routing information,
- selective forwarding,
- sinkhole attacks,
- Sybil attacks,
- wormholes,
- HELLO flood attacks,
- acknowledgment spoofing.

#### 2.1.1. Spoofed, Altered, or Replayed Routing Information

This attack targets the information of a routing exchanged between the nodes. Adversaries are able to establish routing loops, produce false messages, maximize end-to-end latency, and extend or reduce source routes, network partition, and more.

#### 2.1.2. Selective Forwarding

In this threat, malicious nodes may decline to forward particular messages and basically drop them. It makes sure that the malicious nodes are not propagated further as it behaves like a black hole; further all the received messages are rejected. The selective forwarding attacks are normally more efficient as the attacker is explicitly involved in the path of a data flow.

#### 2.1.3. Sinkhole Attacks

By establishing a metaphorical sinkhole with the adversary at the middle, the attacker's goal is to get all the traffic within certain area via a compromised node. With respect to the routing algorithm, this attack can function by making a compromised node appear attractive to the nearby nodes. Various protocols might try to check the route quality with end-to-end acknowledgements comprising the information of reliability or latency.

#### 2.1.4. Sybil Attacks

In this attack, a single node offers several identities to the other nodes in the network. It can significantly minimize the effectiveness of the fault-tolerant systems. This attack also causes a significant attack to geographic routing protocols. By using this attack, an adversary can be in various places at once.

#### 2.1.5. Wormholes

In the wormhole attack, an adversary in one part of the network can receive messages over a low-latency link and replay them in distinct parts via a tunnel. This attack usually includes two detached malicious nodes, which collude to minimize their distance from each other by replaying packets.

#### 2.1.6. HELLO Flood Attack

This attack is a novel attack introduced against sensor networks, where the nodes can be convinced by the adversary to trust that the adversary is its nearby neighbor. This can possibly transfer the fake information with high transmission power. Many packets request nodes to broadcast HELLO packets by assuming themselves as their neighbor nodes. A node thus reaching such a packet will assume that it is within the radio range of the sender.

#### 2.1.7. Acknowledgment Spoofing

This attack has the objective of proving to the sender that a dead node is still alive or a weak link is strong enough. Herein, an adversary can eliminate information transmitting to these dead nodes or weak links. Also, an adversary can eavesdrop packets addressed to the other nodes and identify which nodes are dead or weak.

Table 1 describes several attacks present in the WSN and their corresponding security mechanisms.

### 2.2. Security Requirements and Challenges in WSNs

WSNs share some common functionalities with a typical computer network as it is a special type of network. It also exhibits several characteristics that are unique to it. In WSNs, the most important requirements for security [4] are listed as follows:

- Data confidentiality: it ensures that no messages in the network are understood by the recipient. Also, it provides privacy for wireless communication channels such as mobile codes, application data, and control message so that overhearing is prevented.
- Availability: it guarantees the service presented either by the entire WSN or by any part of it.
- Authentication: before allowing a limited resource or revealing information, it authenticates the sensor nodes, cluster heads, and base stations.
- Authorization: only authorized nodes comprise a particular activity.
- Integrity: ensures that no message or an entity can be changed as it negotiates from the sender to the receiver.
- Freshness: it implies whether the data is recent and safeguards the network against replay attack.
- Nonrepudiation: it protects from the malicious nodes in order to hide their activities.

Towards design of efficient security solution, there are more challenges in the wireless sensor networks than wired networks. They are listed as follows:

- wireless nature of communication,
- resource inadequacy on sensor nodes,
- very large and dense sensor network,
- unknown network topology,
- dynamic network topology.

## 3. Authentication in Wireless Sensor Networks

Authentication is a process by which the identity of a node in a network is verified and guarantees that the data or the control messages originate from an authenticated source. Various authentication procedures consist of

- one-way authentication,
- two-way or mutual authentication,
- three-way authentication,
- implicit authentication.

### 3.1. One-Way Authentication

Only one message is transmitted from the sender node to the receiver node. This message will be able to create

- sender's identity,
- message that is generated by the sender,
- message that is intended to the receiver,
- message that is not altered during transit.

### 3.2. Two-Way or Mutual Authentication

Both entities can authenticate each other in a communication link. In WSN environments, this scheme not only means the authentication between normal nodes and the base station but also mentions the two counterparts that are secure of each other's identity.

### 3.3. Three-Way Authentication

A third message from the sender to the receiver is sent once the clocks of the nodes cannot be synchronized.

### 3.4. Implicit Authentication

Implicit authentication not only is accomplished as an independent process but also is the byproduct of other processes like key establishment. In WSNs, this type of authentication can minimize both operating complexity and energy consumption.

The authentication issues based on the node deployment are (a) static deployment and (b) dynamic deployment. In the former case, the nodes are static and are vulnerable to replay attacks. Authentication protocols should counteract these issues since the nodes are easily traceable. Some of the issues in the latter case are (a) moving node's reauthentication, (b) node's movement that should be untraceable, (c) message integrity, (d) confidentiality, and (e) node capture and compromise.

## 4. Various Authentication Protocols in Wireless Sensor Networks

This section briefly discusses some of the popular authentication protocol schemes in wireless sensor networks.

### 4.1. Lightweight Dynamic User Authentication Scheme

WSN is deployed in a confined area that is separated into several zones. Using mobile devices, the authorized users can access and communicate with the sensor nodes within the WSN. This scheme [5] consists of three phases:

- the registration phase,
- the login phase,
- the authentication phase.

Initially, a user must register with a name and a password at the sensor gateway node before issuing any queries to the system. After successful registration, the user may submit a query to the WSN system at any time within a predefined period. Depending upon the nature of the application, the predefined time period must be set in a different way. The user needs to restart a new cycle by doing the registration again, while the predefined time period has expired. A dynamic user authentication allows the genuine user to query the sensor data from any one of the sensor nodes. It imposes very less computational load, which can be evaluated using simple strong-password based dynamic user authentication protocols for WSNs [6–8]. This lightweight authentication scheme states that it is secure only against replay and forgery attacks.

An enhanced lightweight user authentication scheme [9] shows that it is susceptible to replay and forgery attacks and also maintains the benefit of [5]. It not only upholds all the benefits but also improves its security by enduring the weakness of the security. The system is divided into four stages: registration, login, authentication, and password-changing. Herein, the registration and password-changing stages are implemented via a secure channel. It possesses several benefits, comprising resistance to both replay and forgery attacks, decreasing the risk of user's password leakage, improved efficiency, and ability of changeable password [10].

### 4.2. Lightweight Trust Model

In order to consume less memory and energy, the lightweight schemes are introduced [11–13]. In collaborative lightweight trust-based routing protocol (CLT), the memory consumption is reduced by the following three steps:

- Initially, the trust is computed as positive integer in the range from 0 to 100. It computes only one byte of memory.
- This scheme does not directly store the computed value of trust in the transaction table.
- The memory consumption is reduced significantly as the trust level consumes only 3 bits of memory.

This scheme also enhances the packet delivery ratio using a trust management system. It significantly decreases the energy consumption by avoiding promiscuous operation mode.

### 4.3. Lightweight Authentication Scheme for WSNs

An authentication and key establishment energy-efficient scheme [14] is a novel and suited system to sensor networks. It requires only keyed-hash functions (HMAC) and encryption algorithms in order to provide message confidentiality and authenticity. Also, it decreases the effects of the resource consumption attack. This scheme consists of the following three phases: (i) key predistribution phase, (ii) network initialization phase, and (iii) authentication protocol. Initially, the first phase is carried out during the manufacturing time of the node. Herein, a network-wide symmetric master key is generated and securely stored by the network manufacturer. The second phase takes place during the deployment of the network in which every node discovers its neighbors within the range of communication and sets up the security of the network. Once this phase has been finalized, the authentication protocol takes place every time a new node requests to join the network.

This scheme has a good resilience to node capture attacks, and it scales properly with an arbitrarily huge number of nodes. Similarly, Secured Energy Conserving Slot-Based Topology Maintenance Protocol for Wireless Sensor Networks [15] uses a symmetric key-based authentication mechanism for a sleep/wake-up schedule of nodes for better energy efficiency and increase in lifetime of the network. And the system is proven to be resilient against node capture attack, replay attack, Sybil attack, network substitution attack, and sleep deprivation attacks.

### 4.4. Lightweight Key Management Scheme

In WSN, the lightweight scheme for key management [16] is employed to reduce the resource consumption and acts as the building block for all security mechanisms. This scheme relies on numerical sequences in order to allow each deployed sensor node for estimating distinct pairwise keys with its neighbors. The mandatory objectives are as follows:

- efficiency of resource consumption,
- scalability,
- backward and forward secrecy.

It requires less key storage space with minimal number of message exchanges. The advantages of this scheme are as follows: (a) it occupies less memory space, (b) consumes less energy, and (c) ensures lightweight key computation. Moreover, this scheme will resist node comprising attacks.

### 4.5. SPINS: Security Protocol for Security Networks

According to various requirements of WSN security, SPINS [17–19] offers two kinds of protocol: SNEP and *μ*TESLA to secure communication channels. SNEP protocol offers

- data confidentiality,
- data integrity,
- data authentication,
- freshness of weak message,
- protection of replay message.

A common solution to accomplish message authenticity and integrity is to employ a Message Authentication Code (MAC), which is added along with a message as a signature. The SNEP protocol seems to be feasible for WSN due to the function of the MAC value. The requirements of *μ*TESLA are as follows: (a) base station and sensor nodes should be loosely time synchronized, and (b) each and every node has upper bound information on high time synchronization error. There is a need for more investigations to implement on various modulation approaches of transceiver unit in the sensor nodes. Moreover, the memory must be with maximum computation speed and energy unit.

### 4.6. LEAP: Localized Encryption and Authentication Protocol

In WSNs, LEAP offers multiple keying mechanisms to provide confidentiality and authentication [20, 21]. Based on the different criteria, the packets exchanged by nodes in WSN can be categorized into various classes. Confidentiality may only be essential for some packet types, wherein authentication is vital for all packet types. For each sensor node, LEAP supports the establishment of four kinds of key:

- an individual key shared with the BS,
- a pairwise key shared with the other WSNs,
- a cluster key shared with several neighboring nodes,
- a group key shared by all the nodes in the network.

The authentication scheme known as *μ*-TESLA is employed for the broadcast authentication of the sink node. It ensures that the packets sent with the group are from the sink node only. For source packet authentication, this scheme can also use a one-way hash-key system. In order to establish the above-mentioned four kinds of key, LEAP utilizes a predistribution key. At first, the individual key is established using a function of a seed and the node ID. Furthermore, the nodes broadcast their IDs and evaluate the shared key for the receiving nodes. Then, the cluster key is dispersed by the cluster head using the pairwise shared key. Finally, the network-wide group key is distributed by distributing the sink node in a multihop cluster-by-cluster manner.

Depending on the use of one-way key chains, LEAP consists of an efficient protocol for local broadcast authentication. It may protect or maximize the difficulty of introducing several security attacks in WSNs. In LEAP, the storage requirements per node are small and the procedures for establishment and updating of key are efficient. The major benefits of the LEAP protocol are as follows: (a) comprising *μ*-TESLA, one-way key chain authentication, key revocation, and key refreshing, (b) scalability, and (c) being able to accomplish cluster communications. The drawback of this scheme is that it assumes that sink node is never compromised.

### 4.7. Efficient Authenticated Key Establishment Protocols

In this protocol [22–24], the Elliptic Curve Cryptography (ECC) is employed for performing the security functions on WSNs with inadequate computing resources. Only smaller key lengths are required with ECC for providing a desired level of security than the other public key crypto algorithms. It also offers high processing speed, low computational complexity, and smaller key storage requirements. A self-organizing algorithm using ECC consists of two phases:

- implicit certificate generation process,
- hybrid key establishment process.

The impersonation attack is prevented by using certificates in the key establishment protocol. Here, a certificate is the simple key along with the device identity and certificate expiry date. This scheme has the major difficulty where each node must have direct communication with the CA that might be a bottleneck. The authors did not state the dynamic node reauthentication as well.

### 4.8. Authentication and Key Establishment in Dynamic WSNs

The preshared key-pair is not always present among the roaming nodes and new nearby nodes in dynamic WSNs. Consequently, it necessitates an efficient and scalable protocol for establishing and updating the keys between nodes for secure communication. Every sensor node maintains a table, namely, key cache to manage the keys. The procedure of key management [25] is as follows:

1. Check whether there is an existing key pair among the sensor nodes.
2. If not, process the subroutine of shared-key detection.
3. The sensor node allots an entry in the key cache if there is no common key among them.
4. Once the notice message is received, the session key is recalculated, and the sensor node updates the key stuff and key lifetime.
5. The dynamic sensor node should reinitiate this procedure while the lifetime of the key expires.
6. In order to save the storage, the sensor node eliminates the related entry from its cache table.

Thus, this efficient and scalable protocol is suitable for both the static and dynamic environments. This scheme has maximum probability of sharing a key and less communication cost.

### 4.9. Broadcast Authentication in WSNs

There are two general methods for broadcast authentication in WSNs: digital signatures and *μ*TESLA-based methods [26]. The procedure for these two methods is similar except for the broadcast authenticator generation. Due to their difference in offering the immediate authentication, the procedure for receiving broadcast packets differs slightly for these methods. After the weak authenticator, each receiver can immediately authenticate the signature in signature-based broadcast authentication, whereas the *μ*TESLA-based broadcast authentication does not offer immediate authentication. A dynamic window system has the damage of DoS attack in the minor portion of the nodes. It permits each single node to make its own decision on whether to transfer a message first or check it first. This system [27] is efficient and does not produce too much delay on broadcast.

In order to overcome the difficulty of [27], which is not efficient against the malicious node attack, a group key mechanism [28] was established with the neighbor nodes to resolve the malicious node attack.

The most suitable WSN applications are self-healing key management schemes with broadcast authentication [29]. It is employed to strengthen the security level and also minimize the resource consumption. Moreover, the performances such as security, adaptive sliding window size, and the configurability of self-healing capability will be explored.

### 4.10. Short-Term Public Key System for Broadcast Authentication

This system minimizes the time of signature verification using several short-lived public keys [30]. This scheme uses short length public or private keys, which will minimize the security strength of the public keys. Also, it limits the lifetime of short public keys over the traditional methods that employ one long key. The broadcast authentication becomes less expensive with regard to the short public keys. All the public keys cannot be preloaded into the memory of the sensors due to the memory limitation. In this approach, the problem of original message broadcast authentication is minimized to the problem of public key distribution. The existing progressive public key distribution system is secure, efficient, and resilient to packet loss. Thus, the sink node occasionally broadcasts and reallocates the public keys once the lifetime of these keys expires.

### 4.11. Multiuser Broadcast Authentication

Four various public key-based methods are proposed to offer in-depth analysis of its benefits and limitations [31]. The users are always verified via the public keys in all these methods. The methods are

- a straight-forward certificate-based method,
- direct storage-based system,
- bloom filter-based system,
- hybrid system.

A multiuser authentication scheme is employed for storing user IDs and public keys using bloom filter. The drawback of bloom filter is that it can be forged, and it cannot protect the DoS attack.

### 4.12. Lightweight One-Time Signature Scheme

In WSNs, this scheme allows sensor nodes to authenticate broadcast message from the BS. The symmetric cryptographic primitives are used to accomplish the asymmetric property for broadcast authentication [32]. The general limitations of one-time signature schemes are (a) the use of extremely large key size and (b) the restriction to authenticate only few messages. This scheme efficiently minimizes the requirement of storage and comprises a rekeying mechanism to sign further messages.

- Initially, the signer must generate the pair of keys that consist of private key with private balls and public key with public balls.
- Based on the public balls, the private balls can be authenticated by a verifier.
- There are three phases present in this scheme: initial phase, signing phase, and verification phase.
- The sender produces the private key and its respective public key in the initial phase.
- A pseudo random generator generates a private key that is made of random numbers.
- The public key generation algorithm produces the public key that has hash values, and the sender employs the private key in the signing phase.
- In the verification phase, the receivers utilize the public key of the sender for validating the signature of the message.
- The signature scheme consumes less storage, less communication overhead, and high computation cost when compared to the HORS system.
- This scheme also employs few extra hash computations since storage is a more expensive resource than computation power in a sensor node.

This scheme has four major benefits over *μ*TESLA: no requirement of time synchronization, no buffering needed by a receiver, individual message authentication, and instant message authentication. Moreover, it can improve the strength of the security in terms of very low performance.

### 4.13. Mutual Authentication and Key Establishment Protocol

This system is described for IP-enabled WSN based on 6LoWPAN [33]. The usage of key predistribution methods could not characterize the most accurate solution as the number of hosts in a network varies a lot. Thus, the ECC approach is introduced to maintain a greater security level compared to the other traditional encryption approaches. In order to minimize the total communication overhead and also to avoid the introduction of new vulnerabilities, the joining network authenticates the incoming node easily by creating its authentication key. The major functionalities of this scheme are as follows:

(i) Offline key assignment: a random number and single share of the public key are assigned to each entity of the network. The source and destination IP are employed for generating a particular ECC while considering the secure communication among two nodes in the network.
(ii) Authentication: it allows a trusted node for accessing the network resources.
(iii) Private key generation: the private key is generated as follows:
  (1) Private  key=Public  key⊕Random  number−1mod PSN.
(iv) Handover: it updates the private and the public keys of the nodes to avoid the node replication and Sybil attacks.

This system provides better results against several attacks and also takes less time for exchanging the key establishment packets. Furthermore, the Cooja can be analyzed for total energy consumption and overhead during the connectivity and handover.

### 4.14. EIBAS: An Efficient Identity-Based Broadcast Authentication Scheme

The network of this scheme includes a fixed sink, network users, and a huge number of sensor motes. The sink that serves as a private key generator is liable to generate the private keys for users. It also has limited storage capacity. EIBAS scheme [34] is designed to satisfy the requirements of security and performance: (a) user authentication and message integrity, and (b) reduction of communication overhead. The major contributions of this scheme are as follows:

- System initialization: at first, it generates a prime generator, and a bilinear pairing by the given security parameter. Then, a random number and four cryptographic hash functions are selected.
- Private key extraction: the private key that is generated by the sink should be obtained for the user along with an identity in order to join the WSN.
- Signature generation and message broadcast: initially, it picks a current timestamp, and then the user broadcasts the message in the sensor networks.
- Broadcast authentication: each sensor node checks its authenticity upon receiving the message. Once the verification process fails, the sensor node rejects the message. Or else, the authenticity of the received message is assured.

The pairing-optimal ID-based signature scheme is used to reduce the communication and computational costs. Among all the existing schemes, EIBAS method requires the shortest size of the broadcast message. Also, it minimizes the total energy consumption. Further, it can enhance the overall energy consumption with respect to the size of the network.

### 4.15. Lightweight Authentication Scheme

The lightweight authentication schemes [14, 35] are composed of key establishment and authentication protocols. Herein, the former protocol is carried out during the network deployment. The latter protocol is employed if a new node joins the network while the prior phase is completed. This scheme is efficient, with very lightweight, and does not impose any particular requirement on the network. The solution mentioned in this scheme includes three phases:

- Key predistribution: this phase is carried out before the network is deployed, that is, during the installing time of the node.
- Network initialization: this phase is the initial step for setting up the security of the network, and it is accomplished during the deployment of the network.
- Authentication: this process is carried out every time a new node requests to join the network as the earlier phase has been completed.

The advantage of this system is that it provides (a) a perfect resilience against node capture and (b) node-to-node identity authentication. This system is designed to require only one message to be exchanged, and, thus, it can be further investigated.

Another lightweight authentication scheme is TinyZKP [36], which is designed to verify the sensor node identity of wireless body area network. This system obtains minimum energy consumption and memory consumption and also runs at faster time. It can be implemented in resource-constrained embedded system.

### 4.16. LOCHA: A Lightweight One-Way Cryptographic Hash Algorithm

A lightweight hashing system [37] is described to generate a relatively short-length and fixed hash digest from an input message. The procedure of this scheme is as follows:

- Initially, the input message is preprocessed by converting it into binary ASCII codes.
- It employs padding in the least significant position of the message to make it divisible by 512.
- If the length of the message is already a multiple of 512, then add an extra 512 zeros for improving the robustness of the algorithm.
- Thus, the preprocessed message is divided into 3 levels in a nested manner, which results in 512-, 64-, and 8-bit blocks, respectively.
- Then, the transformations take place for three nested levels to ensure the uniformity and also to minimize the storage overhead.
- The 3-level swapping is applied to receive the final hash digest.

This scheme shows that it is lightweight with respect to the communication, computation, energy efficiency, and storage overhead. It can further employ the generated hash digest in the node or message authentication in wireless sensor network.

### 4.17. Constrained Function-Based Message Authentication

CFA scheme [38] is introduced to support the functionality of the en-route filtering directly as a hash function. It acts as a building block for the other security mechanisms. CFA-based en-route filtering is proposed to defend against false data injection, PDoS attack, and FEDoS attack. This scheme comprises three phases:

- Node initialization: a maximum number of compromised nodes are selected first, and then the adversary can inject falsified data without being detected if it exceeds the global security parameter.
- Report endorsement: a node enters this phase once it has an event report to be sent after sensor deployment. If nodes want to send an event report to the destination node, it first broadcasts an even report in the form of plaintext to its nearby nodes.
- En-route filtering: once the packet is received, the intermediate node verifies whether the attached endorsements are established by the distinct nodes. Once the verification fails, the packet will be dropped.

CFAEF has low filtering capability when compared to the other existing methods. Furthermore, various vulnerabilities can be analyzed numerically and theoretically based on the CFAEF scheme.

### 4.18. Node Level Security Policy Framework

This framework is employed to apply a security policy towards the WSNs [39]. It leverages the properties of the authentication of node and group-based keys for strengthening the network security. In order to provide node authentication and intergroup communication, a group-based key establishment method with identity-based cryptography is used. In order to overcome the node compromise attack, each group of nodes is deployed by prekeying a unique shared key for establishing pairwise communication among the nodes in the network. Thus, the nodes containing the key information for a particular group are allowed to establish pairwise key for future communications. The following phases are a successful WSN deployment:

- Initialization of provisioning authority (PA): an individual PA is generated for each group of nodes to be deployed. A master PA is responsible for creating the pairing information for each PA.
- Initialization of sensor key: each group and every node have a unique identity. Each node in the group is preloaded with the public information for group along with the unique identity-based key.
- Deployment of sensor: the sensors are deployed in groups over the intended area in a predetermined pattern. There is no need of additional bootstrapping for nodes to generate pairwise keys.
- Establishment of pairwise key: it can be established in either of the two ways: intragroup key establishment or intergroup key establishment.

It is resistant to node replication, Sybil, and wormhole attacks in WSNs. Furthermore, it may include multihop key establishment to improve the network capabilities.

### 4.19. Public Key Cryptography-Based Broadcast Authentication Scheme

This scheme [40] is proposed using signature amortization for WSNs that meet the following properties:

- low overhead,
- strong authenticity,
- immediate authentication,
- no time synchronization,
- resilience to node compromise attacks.

This scheme exploits one ECDSA signature for authenticating all broadcast messages. The authenticator in the extended block 0 is employed to authenticate the extended block 1. It contains the broadcast messages and only one authenticator. The process continues until *k*-extended blocks. The overhead of the signature is amortized over all broadcast messages with only one authenticated signature.

This scheme retains greater security besides low overhead and overcomes the defect of *μ*TESLA. This system can accomplish immediate authentication and does not require time synchronization.

### 4.20. AuCRB: Secure Broadcast Authentication Scheme

The security of transferring the broadcast data becomes significant for the networks in hostile areas. Comprising a limited nodes of the network, an adversary initiates serious attacks against the network with high probability of node compromise. Byzantine attackers are considered with the similar authority as any other legitimate node. To prevent the attacks, the cryptographic services are required. Thus, the Authenticated Collaborative Rateless Broadcast (AuCRB) [41] is reintroduced for WSNs. The major contribution in this scheme are as follows:

- Based on a broadcast protocol, AuCRB is designed using rateless coding. Thus, it provides low communication and computation overhead.
- Instead of waiting for multiple packets, the nodes individually authenticate each received packet in order to perform authentication.
- Consequently, the receivers can immediately filter out bogus packet and also save energy.
- The malicious nodes in the network can be detected using the authentication information transmitted by the source.
- In the presence of the malicious nodes, it ensures data availability with very low latency.
- An adversary can compromise nodes and then inject bogus packets or mounts routing attacks by dropping or modifying the packets.
- Moreover, the scheme can be used while the packets are lost due to reasons other than the Byzantine attacks.

### 4.21. Reversible Watermarking Authentication Scheme

A simplified WSN in this scheme has three kinds of nodes: sensor node, transmission node, and sink node. At first, the sensor node groups the data and is composed of two nonoverlapping authentication groups. From the first data group, the watermark bits are computed and embedded into the next before transmission. Furthermore, sink synchronizes the data group and checks the watermark bits from computing and extraction. Lastly, the original data is restored.

In this scheme [42], the dynamic grouping is adopted for ensuring that the number of elements is variable in each group. The watermark generation and embedding consist of the following processes:

- encoding,
- initialization,
- generating,
- embedding,
- decoding.

A new reversible watermarking authentication scheme is employed to verify the integrity and to restore the original data. After watermark embedding, the sensor nodes immediately transmit the data packet, and, thus, the delay will not affect the real-time stream. This technique has no communication, computation, and storage overhead. Further, it can be incorporated with the other techniques for better performance.

### 4.22. Key Management Scheme

In WSNs, key management is an important challenging issue as in [43–45]. The most important contributions of a resilient key establishment protocol are listed as follows:

- distributing the keys in a dynamic method before deployment,
- a dynamic authentication and key establishment methodology with the modules as follows:
  - key predistribution, which is the step based on Elliptic Curve Cryptography (ECC),
  - pairwise key agreement establishment, which permits a node to discover its neighbors and also for establishing secure paths with an authentication phase.

While minimizing the communication overhead and energy consumption, this scheme ensures an enhanced security level. It also resists against compromise node.

## 5. Discussion on Various Protocols

Several authentication protocols for secure wireless sensor networks are depicted. The result of the survey is shown in Table 2. The authentication protocols enhance the security and save energy in WSNs. From the survey, it is evident that a secure lightweight scheme for user authentication and key agreement in multigateway-based WSNs can result in better performance than the existing mechanisms such as MAC-based authentication protocols, key management protocols, and lightweight authentication protocols. Moreover, the surveyed result evidently proves that the incorporation of LEAP protocols is resilient to the various attacks such as sleep deprivation attack, snooze attack, network substitution attack, and insider (clone) attack.

Table 2 describes the information about various authentication protocols in WSNs. The lightweight system for sensor networks [66] can accomplish the following security aspects:

- It detects the wrong input information in the earlier stage of the login phase.
- It is efficient with respect to the computation and communication complexities during the authentication phase.
- It resists towards insider (clone) attack.

The system can yield better energy consumption, communication overhead, and computation overhead than the other existing protocols.

## 6. Future Proposal 

Several authentication mechanisms and lightweight schemes were compared and analyzed with respect to various parameters [67] as follows and summarized in Table 3.

### 6.1. Source Authentication

This parameter is used for the broadcast transmissions that validates the source ID from which the message originates. It is performed by each of the receiver(s) receiving a broadcasted message.

### 6.2. Data Integrity

In data integrity, the content of the message makes sure that it has not been modified during transmission after being transmitted by the sender and before being established by the receivers.

### 6.3. Immediate Authentication

Immediate authentication is accomplished when there is no delay between the message reception and its acceptance/rejection. Most of the MAC protocols do not support this criterion and are not applicable in highly time critical systems.

### 6.4. Time Synchronization

This security condition check helps the receivers by making sure that the respective key has not been released by the sender at the time when a message is received.

### 6.5. Message Cost

The message cost includes the total number of messages required for authentication. If there are more number of message exchanges, the message cost will be high and vice versa.

### 6.6. Communication Overhead

Most of the sensor networks and vehicular networks using MAC based protocols require low communication overhead, whereas the digital signature (DS) based protocols are influenced by the public key size.

Depending upon the message cost, the communication overhead is determined for the authentication protocols. The protocols such as TESLA, *μ*TESLA, multilevel *μ*TESLA, BABRA, unbounded key chains, L-TESLA, X-TESLA, TESLA++, and RPT have low communication overhead as it has the message cost of either 2 or 3. The hierarchical key chains and lightweight schemes use only one message for authentication thereby making the communication overhead very low.

### 6.7. Computation Overhead

The sending side suffers from more computation overhead whereas the receiver computation overhead is negligible. Authentication increases computation overhead that is accountable in both signature generation and verification.

The protocols such as TESLA, *μ*TESLA, multilevel *μ*TESLA, BABRA, L-TESLA, X-TESLA, TESLA++, and RPT have low computation overhead as it uses MD5 cryptographic method by having linear computational complexities. Unbounded key chains use SHA-1 method and it has medium computation overhead due to numerous message exchanges. Hierarchical key chains and lightweight scheme also uses SHA-1 method and requires very low communication overhead due to infinite key chain.

### 6.8. Cryptographic Method

It uses either symmetric key MAC systems or asymmetric key DS systems, wherein the DS systems can be either one time systems or public key based systems. The names of the specific symmetric or asymmetric approaches used in the protocols are mentioned in the Table 3.

### 6.9. DoS Attack Resistance

A protocol is considered as DoS resistant as it offers a countermeasure for one or more of the DoS attacks such as flooding and jamming. It is necessary to make sure that the broadcast authentication protocol executes its activities without interruption.

### 6.10. Robustness to Packet Loss

It is used in terms of loss of authentication information. Most of the TESLA-based schemes use one-way key chains where, once a key is lost, it can be recovered from future keys. It is robust and does not require separate authentication packets.

Based on the above discussion, the future direction of our research incorporates a secure lightweight scheme [14] for sensor networks. This system can accomplish the following security aspects:

- It uses symmetric cryptography with minimum encryption using hash functions.
- It provides node-to-node identity authentication.
- It is efficient with respect to the computation and communication complexities during the authentication phase.
- It resists towards insider (clone) attack with denial of service attacks.

The proposed system will yield better energy consumption, communication overhead, and computation overhead than the existing mechanisms.

## 7. Conclusion 

Security is the major concern for the energy-constrained WSN due to the broad security applications. In recent years, security has attracted a lot of attention and it is very challenging to design strong security protocols. Several schemes proposed on authentication are analyzed to accomplish confidentiality and authenticity of nodes. Most authentication mechanisms focus only on security, while others offer proper scalability, minimized communication, and computation overhead. The authentication is an efficient methodology to repel various attacks as it requires sharing of keys. It is therefore evident from the literature that an authentication scheme can reduce the computation cost and save energy. Based on our comparisons and study, we conclude that authentication mechanism has been widely used nowadays but still suffers from the following issues such as complex management of public key infrastructure and computational bottleneck which have to be resolved by future research.

## Figures and Tables

**Table 1:** Several attacks and their corresponding security mechanisms in WSN.

Type of attack	Layer	Security mechanism
Jamming	Physical	(1) Lower duty cycle
		(2) Spread-spectrum technique

Tampering	Physical	(1) Key management schemes

Collision	Data link	(1) Error correcting code

Exhaustion	Data link	(1) Rate limitation

Replayed routing information	Network	(1) Encryption techniques
		(2) Authentication schemes

Selective forwarding attack	Network	(1) Redundancy technique
		(2) Probing mechanism

Sybil attack	Network	(1) Authentication schemes

Sinkhole attack	Network	(1) Authentication schemes
		(2) Redundancy technique
		(3) Monitoring

Wormhole attack	Network	(1) Flexible route selection method

HELLO flood attack	Network	(1) 2-way authentication method
		(2) 3-way handshake method

Flooding attack	Transport	(1) Minimizing connection numbers
		(2) Client puzzles

Clone attack	Application	(1) Unique pairwise keys

**Table 2:** Information about different authentication protocols in wireless sensor networks.

Techniques	Author and reference	Year	Performance	Quality measurement
Lightweight authentication protocols				
Lightweight authentication protocol (LAP) for smart dust WSNs	Sharifi et al. [46]	2009	LAP employs comparatively fewer keys to accomplish security for nodes before deployment and minimizes the communication overhead	(1) Less computational requirements (2) Less communication requirements (3) Less overhead
Lightweight authentication scheme for WSNs	Delgado-Mohatar et al. [14]	2011	This scheme employs symmetric cryptography and encryption algorithm to provide perfect resilience against various attacks	(1) Smaller number and length of the exchanged messages (2) Low power consumption (3) Better scalability
Lightweight authentication for recovery in WSNs	Li et al. [47]	2009	This scheme is used to recluster and reprogram the nodes in a WSN	(1) Low execution time (2) Minimum number of verifications
Lightweight protocol	Shah et al. [48]	2014	This protocol utilizes Fermat Number Transform (FNT) and Chinese Remainder Theorem (CRT) for enabling secure communication	(1) Minimum memory utilization (2) Data confidentiality (3) Anonymity (4) Instant authentication (5) Mutual authentication (6) Data integrity (7) Data freshness
LSec: Lightweight Security protocol for WSN	Shaikh et al. [49]	2006	LSec offers authentication and authorization of sensor nodes. Also, it provides simple key exchange scheme and data confidentiality	(1) Less memory requirement (2) Low transmission cost
Lightweight security framework	Zia and Zomaya [50]	2011	This mechanism ensures a sensor node to base station and also has better total security for WSNs	(1) Packet transmission time (2) Low latency (3) Less packet overheads
Self-key establishment protocol for WSNs	Sharifi et al. [51]	2009	SKEW uses a refreshing mechanism for offering greater security. It does not need a particular key server for key broadcasting	(1) Less communication overhead (2) Reducing energy consummation (3) Less memory usage (4) Scalability (5) Local connectivity (6) Global connectivity

Key management protocols				
LEAP: localized encryption and authentication protocol	Zhu et al. [20]	2006	Based on the use of one-way key chains, LEAP comprises an efficient protocol for local broadcast authentication. It maximize the difficulty of introducing various security attacks on WSN	(1) Low computational cost (2) Low communication cost (3) Less storage requirement
BROSK: broadcast session key	Camtepe and Yener [52]	2005	BROSK uses master key for establishing session key. It is the master key based key distribution solutions	(1) Less memory requirements (2) Very low resilience
LKHW: logical key hierarchical for wireless sensor networks	Pietro et al. [53]	2003	LKHW offers secure multicasting using an extension of the directed diffusion protocol. It also supports both backward and forward secrecy	(1) Robustness in routing (2) Robustness in security
Random key distribution scheme	Du et al. [54]	2004	This scheme uses the deployment knowledge and accomplishes the level of connectivity. It also enhances the resilience of the network against node capture	(1) Less communication overhead (2) Network resilience
Pairwise keys in sensor networks	Liu et al. [2]	2005	This system enables sensor nodes to communicate securely with each other via the cryptographic methods	(1) Resource constrained (2) Low storage (3) Low communication overhead (4) Low computation overhead

MAC-based broadcast authentication protocols				
Multiple TESLA	Perrig et al. [55]	2005	This protocol addresses the scalability of TESLA minimizing the congestion load using distributed and secure time servers	(1) Low space overhead (2) Less authentication delay
*μ*TESLA	Ullah et al. [56]	2011	This protocol saves energy by minimizing the size of transmitted packets	(1) High computation power (2) High communication bandwidth (3) Less memory requirements
Multilevel *μ*TESLA	Liu and Ning [57]	2004	This scheme offers a solution for the unicast bootstrapping problem of *μ*TESLA. It also makes broadcasts scalable to a new receiver	(1) Fault tolerance (2) DoS tolerance (3) Less computation overhead
Scalable *μ*TESLA	Liu et al. [58]	2005	This scheme improves scalability by maximizing the number of senders. For the distribution of initial parameters and commitments, the Merkle hash tree is used in *μ*TESLA	(1) Time synchronization (2) Less storage overhead
Regular predictable TESLA (RPT)	Luk et al. [59]	2006	RPT offers an immediate solution to the authentication delay problem	(1) Time synchronization
BABRA	Zhou and Fang [60]	2006	This scheme is based on *μ*TESLA symmetric key broadcast authentication mechanism using delayed key disclosure. It uses the similar batch key for all messages transmitted during a specific communication period	(1) Time synchronization (2) Infinite number of keys (3) Low packet loss
Unbounded one-way chains	Groza [61]	2008	This scheme overcomes the limitation of length of key chains in standard TESLA using squaring function	(1) Scalability (2) Reliability (3) Less bootstrapping overhead
Long duration TESLA	Liu et al. [62]	2012	This protocol modifies the creation of the key chain and also overcomes the limited length of one-way key chain used in *μ*TESLA	(1) Less execution time
TESLA++	Studer et al. [63]	2009	In this protocol, only the MAC of the message is broadcast with the index number of the recent key	(1) Less memory/space requirement
Localized TESLA (L-TESLA)	Drissi and Gu [64]	2006	This minimizes the authentication delay by partitioning a large network to multiple smaller subsets	(1) Low verification delay (2) Less broadcast overhead (3) Low broadcast delay
Extended TESLA (X-TESLA)	Kwon and Hong [65]	2010	The major purpose of this protocol is to save energy and avoid data-memory trade-off attacks	(1) Reducing memory consumption

**Table 3:** Comparison of existing and lightweight authentication schemes.

Authentication protocols	Source authentication	Data integrity	Immediate authentication	Time synchronization	Communication overhead	Computation overhead	Cryptographic method	DoS Resistance	Robustness to packet loss	Message Cost
TESLA	Yes	Yes	No	Yes	Low	Low	MD5	No	Yes	2
*μ*TESLA	Yes	Yes	No	Yes	Low	Low	MD5	No	Yes	3
Multilevel *μ*TESLA	Yes	Yes	No	Yes	Low	Low	MD5	Yes	Yes	3
BABRA	Yes	Yes	No	No	Low	Low	MD5	Yes	Yes	3
Unbounded key chains	Yes	Yes	No	Yes	Low	Medium	SHA-1	No	Yes	2
L-TESLA	Yes	Yes	No	Yes	Low	Low	MD5	No	Yes	3
X-TESLA	Yes	Yes	No	Yes	Low	Low	MD5	Yes	Yes	3
TESLA++	Yes	Yes	No	Yes	Low	Low	MD5	Yes	Yes	2
RPT	Yes	Yes	No	Yes	Low	Low	MD5	No	Yes	3
Hierarchical key chains	Yes	Yes	No	Yes	Very Low	Very Low	SHA-1	No	Yes	1
Lightweight scheme	Yes	Yes	No	Yes	Very Low	Very Low	SHA-1	No	Yes	1
